# Wnt Signaling and Its Significance Within the Tumor Microenvironment: Novel Therapeutic Insights

**DOI:** 10.3389/fimmu.2019.02872

**Published:** 2019-12-16

**Authors:** Sonal Patel, Aftab Alam, Richa Pant, Samit Chattopadhyay

**Affiliations:** ^1^National Centre for Cell Science, Savitribai Phule Pune University, Pune, India; ^2^Department of Cancer Biology and Inflammatory Disorder, Indian Institute of Chemical Biology, Kolkata, India

**Keywords:** immune response, signaling, immunotherapy, β-catenin, anti-tumor response

## Abstract

Wnt signaling is one of the central mechanisms regulating tissue morphogenesis during embryogenesis and repair. The pivot of this signaling cascade is the Wnt ligand, which binds to receptors belonging to the Frizzled family or the ROR1/ROR2 and RYK family. This interaction governs the downstream signaling cascade (canonical/non-canonical), ultimately extending its effect on the cellular cytoskeleton, transcriptional control of proliferation and differentiation, and organelle dynamics. Anomalous Wnt signaling has been associated with several cancers, the most prominent ones being colorectal, breast, lung, oral, cervical, and hematopoietic malignancies. It extends its effect on tumorigenesis by modulating the tumor microenvironment via fine crosstalk between transformed cells and infiltrating immune cells, such as leukocytes. This review is an attempt to highlight the latest developments in the understanding of Wnt signaling in the context of tumors and their microenvironment. A dynamic process known as immunoediting governs the fate of tumor progression based on the correlation of various signaling pathways in the tumor microenvironment and immune cells. Cancer cells also undergo a series of mutations in the tumor suppressor gene, which favors tumorigenesis. Wnt signaling, and its crosstalk with various immune cells, has both negative as well as positive effects on tumor progression. On one hand, it helps in the maintenance and renewal of the leucocytes. On the other hand, it promotes immune tolerance, limiting the antitumor response. Wnt signaling also plays a role in epithelial-mesenchymal transition (EMT), thereby promoting the maintenance of Cancer Stem Cells (CSCs). Furthermore, we have summarized the ongoing strategies used to target aberrant Wnt signaling as a novel therapeutic intervention to combat various cancers and their limitations.

## Introduction

Tumorigenesis is a multifaceted process largely occurring due to the accumulation of mutations in the tumor suppressor genes and oncogenes. These mutations lead to uncontrolled proliferation and resistance to cell death. The sustenance and fate of a tumor is dictated by the tumor microenvironment, which fulfills the needed requirements of energy, growth-factors, chemokines, cytokines, and autocrine/paracrine signals, thereby attracting a wide variety of cell types (1). The tumor microenvironment consists of fibroblasts, immune cells, endothelial cells, and the extracellular matrix. The fine balance between these cells and the transformed cells is decided by the variety of signaling pathways; one such critical pathway is the WNT signaling cascade. Wnt signaling controls a plethora of functions with the help of 19 Wnt proteins, 2co-receptors, 10 Frizzled (Fzd) receptors, and various non-Fzd receptors, for example the Receptor Tyrosine Kinase-like Orphan Receptor and the Ryk Receptor-like Tyrosine Kinase (2). Wnt ligands are secreted lipid-modified glycoproteins and have varied functions that include hematopoietic stem cell maintenance, cell migration, cancer stem cell survival and maintenance, and inflammation and immune tolerance (3–5). These ligand–receptor interactions activates various signaling cascades that are important for cellular homeostasis, oncogenic transformation, tumor progression, and metastasis (3, 6). The Wnt signaling pathway is known to be critical in T cell and dendritic cell development and maturation, which are the epicenter of the adaptive immune response, making it a vital signaling pathway for fighting various pathophysiological disorders (5). Due to its involvement in diverse functions, any aberration or de-regulation of this pathway causes several types of cancers and/or developmental defects. It regulates the anticancer immune response and has shown correlation with poor prognosis and survival of cancer patients. The tumor microenvironment (TME) is a source of both canonical and non-canonical Wnt ligands and can induce aberrant signaling pathways in the cancer cells and immune cells, leading to EMT and altered immune response (7, 8). For several years, it has been established that the Wnt/β-catenin pathway is indispensable for cancer cell survival and maintenance, making it a lucrative target for an anticancer therapy regimen. Various Wnt inhibitors are undergoing clinical trials for therapeutic purposes (alone or in combination with other anti-cancer drugs). This review highlights the role of Wnt/β-catenin signaling cascade in tumor microenvironment and its effects in cancer progression and survival. We conclude by accentuating the potential of Wnt/β-catenin inhibitors in the harnessing of new anticancer therapeutics by targeting cancer microenvironment.

## Wnt Signaling

In 1973, thewingless gene was discovered during a mutagenesis screening for temperature-sensitive mutants in *Drosophila melanogaster* (9). Consequently, many other genetic components involved in embryonic pattern formation were identified (10). The foundation research for Wnt signal transduction was carried out in the 1980s and 1990s, and it was established that the gene products of the Drosophila wingless (wg) and murine proto-oncogene Int1 (now called Wnt1) are orthologous (11). The term “Wnt1” is an amalgamation of *wingless* and *Int1* (12).

WNTs are a large family of secreted, hydrophobic, and Cys-rich glycolipoproteins that direct developmental processes, stem cell proliferation, and tissue homeostasis throughout the metazoans (13, 14). As a result, any abnormality in the Wnt signaling pathway causes pathological conditions such as birth defects, cancers, and other diseases (15). In humans, there are 19 genes encoding WNTs that connect to various receptors and stimulate different intracellular signal transduction pathways (16). Based on different studies, these pathways have been roughly divided into either canonical (β-catenin dependent) or non-canonical (β-catenin independent) signaling pathways (16), as is described in the subsequent section. Depending upon their potential to induce morphological transformation in a murine mammary epithelial cell line (C57MG), the Wnt family has been categorized into different types (17). Wnt1, Wnt3, Wnt3a, and Wnt7a fall under the category of highly transforming members, and Wnt2, Wnt4, Wnt5a, Wnt5b, Wnt6, Wnt7b, and Wnt11 are grouped under intermediately transforming or non-transforming members (13). In general, Frizzled proteins function as common receptors for both canonical as well as non-canonical pathways (16).

### Canonical Wnt Signaling

The canonical Wnt signaling pathway is a well-studied pathway that is activated by the interaction of Wnt with a Frizzled (Fz) receptor and LRP5/LRP6, where LRP stands for lipoprotein receptor-related protein (which is a single-span trans-membrane receptor) (16). Once bound by Wnt, the Fz/LRP co-receptor complex stimulates the canonical signaling pathway. Upon activation, Fz can interact with a cytoplasmic protein called Disheveled (Dsh), which acts upstream of β-catenin GSK3β (15). Research studies have identified Axin as a protein that interacts with the intracellular domain of LRP5/6 through five phosphorylated PPPSP motifs in the cytoplasmic tail of LRP (18, 19). GSK3 phosphorylates PPPSP motifs, whereas Casein kinase 1-γ (CK-1γ) phosphorylates multiple sites within LRP5/6, which in turn promote the recruitment of Axin to LRP5/6. CK-1γ isoforms within the CK-1 family carry putative palmatoylation sites at the carboxy terminal (20).

**Figure 1:**
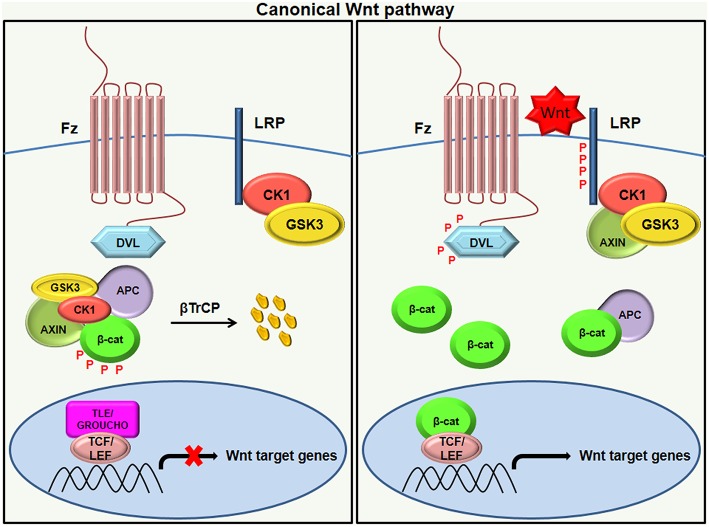
Canonical Wnt signaling. In the absence of a Wnt ligand (left), the phosphorylation of β-catenin by destruction complex (composed of axin, APC, CK1, and GSK3β) leads to its ubiquitination by β-TrCP targeting it for proteasomal degradation. The absence of β-catenin in the nucleus results in the binding of the repressor complex containing TCF/LEF and TLE/Grouche to the target gene and thereby repressing its activity. Once the Wnt ligand binds to the Frizzled receptor and LRP co-receptor (right), LRP receptors are phosphorylated by CK1 and GSK3β, resulting in the recruitment of Dvl proteins to the plasma membrane where they activate and scaffold the β-catenin destruction complex. This results in the accumulation of β-catenin in the cytoplasm and its translocation to the nucleus where it forms a complex with TCF/LEF and transcribes target genes.

In unstimulated situations when Wnt is inactive, the transcriptional co-activator β-catenin is rendered inactive due to its phosphorylation by GSK-3. Inactivation of β-catenin is characterized by the formation of a “destruction complex” that comprises of GSK3, adenomatosis polyposis coli (APC), Axin, and casein kinase Iα (CKIα) (16). This destruction complex leads to the ubiquitination of β-catenin by an E3 ubiquitin ligase called β-TrCP and targets it for proteasomal degradation (21). As a result, β-catenin is not translocated to the nucleus and the repressor complex containing T-cell specific factor (TCF)/lymphoid enhancer-binding factor (LEF) and transducing-like enhancer protein(TLE)/Grouche binds and represses the activity of the target gene (14, 22, 23). Following the binding of Wnt to Frizzled-Axin-LRP-5/6 complex, cytosolic GSK-3β (Glycogen synthase kinase-3 beta) is sequestered, and the phosphorylation of β-catenin is blocked. The accumulation of hypo-phosphorylated β-catenin in the cytosol allows its migration to the nucleus, where it regulates target gene expression by interacting with the TCF/LEF family of transcription factors (Figure 1). This signaling is implicated in the regulation of cell differentiation and proliferation (3, 24).

### Non-Canonical Wnt Signaling

**Figure 2:**
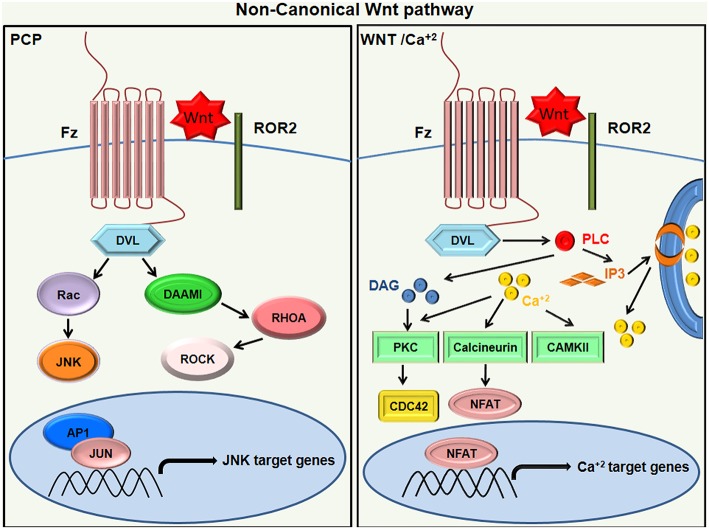
Non canonical Wnt signaling. In Wnt/PCP signaling (left), the binding of Wnt ligands to ROR-Frizzled receptor complex results in the activation of Dvl. The activated Dvl triggers the activation of small GTPase Rho by the de-inhibition of cytoplasmic protein DAAM. Rac1 and Rho together trigger ROCK and JNK and promote polarized cell migration. On the other hand, the WNT/Ca+2 pathway (right) activates PLC to produce DAG and IP3, leading to intracellular calcium fluxes that activate PKC isoforms other than calcineurin and calcium-modulated kinases (CAMKII), which then exhibit an NFAT-dependent transcriptional response.

The β-catenin-independent pathway does not involve β-catenin–TCF or β-catenin–LEF components but utilizes alternative means of downstream signaling, which may elicit a transcriptional response. These pathways are categorized depending on the type of Wnt receptor and co-receptor they employ and the downstream receptors they pair with (16). The non-canonical signaling majorly activates PCP, RTK, or Ca^+2^ signaling cascades through FZD and/or ROR1/ROR2/RYK co-receptors (25). The typical example of the β-catenin independent pathway is the PCP signaling pathway. Human non-canonical WNTs generally include Wnt5A, Wnt5B, and Wnt11, which transduce PCP (Planar Cell Polarity) signals through the receptors; FZD3 or FZD6; and co-receptors ROR1, ROR2, or PTK7 (26). In the PCP pathway, the Frizzled receptor activates a cascade involving a small Rho family of GTPases (Rho, Rac, and Cdc42) and Jun-N-terminal kinase (JNK) [Figure 2; (27, 28)]. The PCP pathway is involved in regulating cell polarity during morphogenesis. Another example of β-catenin-independent signaling is the Wnt-Ca^+2^ pathway. NFAT (nuclear factor of activated T cells) and TAK1-induced Nemo-like Kinase (NLK) are calcium regulated transcription factors of the non-canonical pathway (29, 30). The binding of Wnt ligand to a Fz receptor results in the activation of phospholipase C, which is located on the plasma membrane of the cell. This, in turn, stimulates the production of certain signaling molecules, such as diacylglycerol (DAG) and 1, 4, 5-triphosphate (IP3). IP3 triggers the intracellular release of Ca^+2^ ions and activation of effector molecules like protein kinase C (PKC), calmodulin-dependent kinase II (CAMKII), and calcineurin. This consequently activates the transcriptional regulator NFAT (Figure 2). The Wnt/Ca^+2^ pathway is implicated in cancer, inflammation, and neurodegenerative diseases (31).

## Tumor microenvironment

The cooperative interaction between cells and their microenvironment is imperative for normal tissue homeostasis as well as for tumor growth (32). The tumor microenvironment (TME) has a pivotal role in modulating the metastatic properties of cancer cells and, thus, cancer progression. A range of stromal cells in the adjoining environment are recruited to tumors and facilitate the metastatic distribution to even the most remote organs (33). Solid tumors are not just the arbitrary combination of cells and the extracellular matrix (ECM), they also include adjoining blood vessels, i.e., vasculature and multiple cell types (fibroblasts, endothelial, immune cells, etc.), signaling molecules, and ECM components, which develop complex interactions and start utilizing processes that are similar to those used by developing organs (34). In addition to the abovementioned components of TME, some other components also exist; for example, adipose cells and neuroendocrine cells within the tumor microenvironment also have an important role in tumor aggression and invasiveness. Recent developments in tumor immunology and immunotherapy have described IL-8 as a potent biomarker in different tumors (35, 36). This may have a profound effect on the tumor microenvironment as IL-8 receptor expression is not only found in cancer cells but also in endothelial cells, TAMs, and neutrophils (37). The function of different cells and their markers, along with the aforementioned components, are compiled in the tabular form (Table 1).

**Table 1:** Components of the tumor microenvironment: main markers and their key functions.

**S. no**.	**Component of TME**	**Main markers**	**Key functions**	**Reference(s)**
1	Vasculature	Vascular endothelial growth factor (VEGF), CD31, CD34, Placental growth factor (PlGF), Platelet derived growth factor-β (PDGF-β), TGFα	Blood vessel formation and nutrient and oxygen supply. Evacuate metabolic waste and CO_2_. Help to escape immune surveillance	(38–40)
2	Cancer associated fibroblasts (CAFs)	Epidermal growth factor (EGF), Fibroblast growth factor (FGF), MMP2, CXCL12, CXCL14, Hepatocyte growth factor (HGF), VEGF, PDGF, stromal cell derived factor-1 (SDF-1) and constituents of ECM (OPN)	Integrate collagen and protein to form the Extracellular matrix (ECM), participate in wound healing, and angiogenesis. Regulate inflammation and escape damage to tissues.	(38, 40–42)
3	Inflammatory cells	HMGB1, Foxp3+, TNF-1α, IL-10, IL-12, IL-6, TGF-β, CD163+, KIR, PD-1+, IL-8, IL-4, IL-19, IL-17	Sustained immunosuppression, clearing cellular debris, and treatment of wound healing and infection. Expression of PD-L1 in TME and activation of NK cells and T lymphocytes	(35, 36, 40, 43–45)
4	Extracellular matrix (ECM)	Collagen, fibronectin, proteaglycans, laminin, laminin, vitronectin, tenascin-C, SPARC	Provides mechanical strength. Makes it difficult for drug to penetrate tumor	(46, 47)
5	Tumor associated endothelial cells (TECs)	VEGFR, EGFR, VEGF, PGE_2_, TGF-β, IL-6 and IL-10, IL-8	Increased proliferation and migration properties, angiogenesis, and immune suppression	(35–37, 48–50)
6	Adipose cells	Aromatase inhibitors (AIs), methyl-CpG-binding protein 6 (MBD6)	Produce circulatory blood estrogen, vasculogenesis, inflammation, fibrosis, source of adipokines (leptin, adiponectin), remodeling ECM, recruitment of immune cells, IL-6, IL-8, CCL2, and COX2	(40, 51)
7	Neuroendocrine cells	Ki-67, IL-2, KE108, Delta-like canonical notch ligand 3 (DLL3), EGF, Chromogranin A (CgA)	Regulate secretion and motility, inflammation, and angiogenesis	(40, 52)

### Wnt Signaling and the Tumor Microenvironment

The epithelial mesenchymal transition (EMT) is an indispensable process throughout morphogenesis in which epithelial cells lose cell–cell contact, polarity, and other properties of epithelial cells and acquire the properties that are distinctive of mesenchymal cells (like increased motility). Characteristic features of EMT include the loss of E-cadherin at the plasma membrane, the gain of vimentin and fibronectin, and the increased accumulation of nuclear β-catenin. The transition from an epithelial to mesenchymal cell type requires a range of inter- or intra-cellular changes. In addition to performing normal developmental processes, EMT also plays a key role in tumor growth and progression if it goes unchecked (53, 54). The tumor microenvironment (TME) plays a crucial role in assisting cancer metastasis by inducing EMT in the tumor cells. Many different signaling pathways are known to be involved in EMT, such as TGF-β, NF-κB, Notch, Wnt, and receptor tyrosine kinase (55). In this section, we discuss the role of TME in the activation of the Wnt/β-catenin signaling pathway and, thus, epithelial to mesenchymal transition.

Besides Wnt ligands, the growth factors secreted by stromal cells of TME are also responsible for the activation of Wnt signaling in the nucleus (56). For example, stimulation of hepatocyte growth factor (HGF) in colorectal cancer cells (CRCs) promotes phosphorylation of β-catenin in tyrosine residue and its dissociation from Met (HGFR is encoded by proto-oncogene MET) and thus upregulates β-catenin expression via the PI3-K pathway. Moreover, augmented HGF levels enhance the activity of the β-catenin-regulated TCF family of transcription factors. Studies suggest that Met and β-catenin also assist the entry of cells into cell cycle and prevent them from undergoing apoptosis. For example, c-Met overexpression is significantly correlated with cervical cancer progression (57). Therefore, the crosstalk between HGF released from TME and Wnt/β-catenin in CRCs encourages tumor growth and invasion (58).

Another growth factor responsible for the activation of Wnt/β-catenin signaling is the platelet-derived growth factor (PDGF). A study by Yang et al. suggests that PDGF treatment led to the phosphorylation of p68 (a member of the DEAD box family of RNA helicases) at Y593 residue in the cell nucleus (59). Y593 phosphorylated p68 promotes the nuclear translocation of β-catenin by blocking its phosphorylation by GSK-3β and dislodging axin from β-catenin. Subsequently, β-catenin interacts with LEF/TCF in the nucleus and initiates the EMT process (59). Likewise, EGF and TGF-β also induce p68 phosphorylation at tyrosine and require p68 for EMT initiation. Therefore, the p-68-β-catenin axis may correspond to a common output for various signaling pathways (54).

The vascular endothelial growth factor (VEGF) is another growth factor that is regarded as the archetype molecule in malignant phenotype (38). VEGF expression and micro-vessel density are regarded as the prognostic factors for poor outcomes in various cancers (60). For instance, the overexpression of VEGFA in aggressive oral squamous cell carcinoma (OSCC) may serve a vital prognostic factor for this kind of cancer (61). In addition to VEGF, a transcription factor known as the ETS-related gene (ERG) belonging to ETS (E26 transformation-specific) family is also implicated in angiogenesis and vascular development. Overexpression of ERG in a mouse model reduces the vascular permeability and increases VEGF-dependent angiogenesis through Wnt/β-catenin signaling. This happens because ERG controls the transcription of Fzd4 receptors and stabilizes β-catenin levels in endothelial cells (62).

Cancer-associated fibroblasts (CAFs) are also known to play a vital role in shaping the immunosuppressive environment within the tumor, specifically in oral squamous cell carcinoma (OSCC), wherein CAF-educated cells suppress the T cell population more efficiently than the control cells (63). CAFs are also considered to be a main source of Wnt2 in colorectal cancer where FZD8 acts as a putative receptor of Wnt2 and is responsible for tumor growth, invasions, and metastasis (64).

Prostaglandin E2 (PGE2) is an effective mitogen that is secreted by TECs of the tumor microenvironment. It activates β-catenin signaling and help in the proliferation of colon cancer cells. Once stimulated, PGE2 can trigger EP2 receptors linked to the heterotrimeric G protein of Gs family. The activated α-subunit of Gs binds to the RGS domain of axin and promotes the dissociation of GSK-3β from its complex with axin. Consequently, free βγ subunits stimulate the activity of PI3K and Akt, thereby resulting in phosphorylation and inactivation of GSK-3β. All these processes lead to translocation of β-catenin to the nucleus and stimulation of growth-promoting genes and, thus, cancer progression (65).

The interaction of inflammatory cells with cancer cells is well-studied. In colorectal cancer, the infiltrating macrophages express high levels of Wnt2 and Wnt5a in progression from normal colorectal adenoma to carcinoma. *In-situ* hybridization studies showed that transcripts of Wnt2 and Wnt5a were majorly present in the lamina propria/stroma region within the macrophages. This suggests that paracrine Wnt activation by macrophages may result in cancer progression (66). Wnt7b is another Wnt ligand that is produced by macrophages residing in the tumor (67). One study proposed a mechanism wherein macrophages produced Wnt7b and initiated canonical the Wnt signaling pathway in vascular endothelial cells (VECs) expressing LRP5 and Frizzled in a paracrine fashion. This ultimately lead to the stabilization of β-catenin and entry of VECs in the cell cycle. In the absence of death/apoptotic signal to VECs, this provided a mechanism for the stimulation of VECs via macrophages and tumor angiogenesis (68). Also, Wnt ligands secreted by tumor cells could stimulate the polarization of TAMs to M2 subtype via the canonical Wnt signaling pathway, resulting in tumor growth and migration (69). Moreover, macrophage-derived soluble factors also induced canonical Wnt signaling pathway and promoted tumor growth and metastasis. For example, tumor cells induced the release of IL-1β from macrophages, thereby inducing the phosphorylation of GSK3β and stabilizing β-catenin. This results in higher expression of Wnt target genes in cancer cells. The constitutive expression of STAT1 in macrophages is required for activation of IL-1β, which is essential in order for these macrophages to induce Wnt signaling (70).

Components of the extracellular matrix (ECM) also regulate tumor cells, particularly of the colon. Wnt ligands are expressed in both epithelial and mesenchymal cells of the colon. Moreover, aberrant Wnt signaling is also associated with the development of colorectal cancer (71). Mesenchymal forkhead transcription factors, *Foxf1* and *Foxf2*, can promote ECM production in the gut and can limit paracrine Wnt signaling. A study by Ormestad et al. suggested a crosstalk between stromal cells and parenchymal cells, which involves Wnt signaling. According to their findings, deletion of *Foxf1* and *Foxf2* resulted in the enhanced expression of Wnt5a in the mesenchymal cells and nuclear translocation of β-catenin in epithelial cells. This resulted in over-proliferation of intestinal cells, which are also resistant to apoptosis (72).

**Figure 3:**
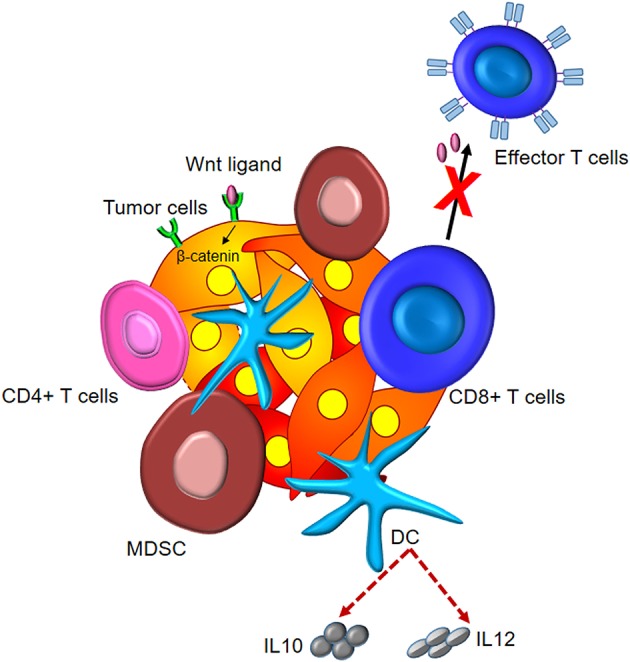
Tumor immunomodulation by Wnt/β-catenin signaling. Wnt signaling induces elevated levels of intracellular β-catenin in malignant cells. This leads to the subsistence of Tregs, differentiation of CD4+ T cells to Th17 subtype, and secretion of IL10 and IL12 by DCs and dampened effector differentiation.

Altogether, these studies bring about the crucial role of different components of the tumor microenvironment in activation of Wnt/β-catenin signaling and, therefore, tumor invasion and metastasis. The effect of Wnt/β-catenin signaling on tumor immunomodulation is depicted in Figure 3.

## Wnt Signaling in Haematopoiesis and immune cell maintenance

WNT signaling works in a context-dependent manner. Under different physiological conditions, it can positively or negatively regulate immunosurveillance mechanisms in the tumor microenvironment. Wnt signaling is an important pathway for immune cell maintenance and renewal. It regulates the progenitor cell homeostasis, thereby controlling hematopoiesis. Various Wnt ligands such as Wnt5a, Wnt10b, and Wnt16 have been reported in regulating hematopoiesis (73–75). Apart from Wnt ligands, downstream molecules of Wnt signaling also control the development and differentiation of various hematopoietic cells. β-catenin is one of the most indispensable molecules for both canonical and non-canonical Wnt signaling and has been shown to positively regulate hematopoiesis, which confers an increased population of hematopoietic stem cells both *in-vitro* and *in-vivo* (76). GSK-3β inhibitors activates Wnt signaling by blocking degradation of β-catenin in murine and human HSCs. *In-vivo* administration of GSK-3β inhibitors has shown potential effects in enhancing the HSCs engraftment during bone marrow transplantation models (77). Besides its role in hematopoiesis, Wnt signaling is also demonstrated to play a role in thymocyte development where it helps in proliferation of immature thymocytes (78). Wnt signaling is involved in consecutive thymic selection events leading to maturation of naive T cells. Wnt inactivation by Dickkopf-related protein 1 (DKK1) in postnatal mice resulted in the loss of progenitor thymic epithelial cells and thymic degeneration (79), highlighting the importance of the presence of Wnt in the microenvironment. Wnt signaling also plays a crucial role in the B-cell development where it controls proliferation and survival of progenitor B-cells. Lef-1-deficient mice showed defects in progenitor B-cell proliferation and survival (80). Recently, it has been shown that the canonical and non-canonical Wnt signaling could contribute to the maintenance and differentiation of B-1 cells, a subpopulation of B cells localized in the peritoneum (81). Thus, Wnt signaling plays a pivotal role in maintenance of hematopoiesis and renewal of immune cells in circulation and thereby positively regulates the anticancer immune response.

### Wnt Signaling Dampens the Antitumor Immune Response in the Tumor Microenvironment

Wnt signaling also play a crucial role in dampening antitumor immune response in tumor microenvironment. There are several reports, which suggest that Wnt signaling encourages tumor progression by promoting tolerance and the immune-escape mechanism. The cumulative anti-tumor immune response mediated by T cells, dendritic cells, B cells, macrophages, neutrophils, and NK cells results in tumor regression caused by elimination of cancer cells (82). Mutations and epigenetic changes developed by tumors due to continuous stimulation by carcinogens or the environmental factors alter the signaling cascade in the tumor microenvironment (1). These changes alter the phenotypic expression of chemokines, cytokines, and some important ligands for the immune cells in the tumor microenvironment resulting in tolerance and immune escape. Recognition and elimination of the cancer cells is largely dependent on antigen presenting cells (APCs), such as dendritic cells (DCs), macrophages, and B cells, which have the ability to present tumor associated antigens (TAAs) (83). Suppressor cells present in the tumor microenvironment, such as Treg, myeloid-derived suppressor cells (MDSC), DC suppressor cells, etc., induce tolerance and tumor progression (84, 85). Wnt signaling has shown a contradictory role in the regulation of MDSCs; on one hand it inhibits MDSC maturation, but, on the other hand, it promotes VEGF expression, which positively regulates MDSCs (86, 87). Also Wnt 5a induces IL12 expression by the dendritic cells altering the suppressive function of MDSCs (88, 89). Despite MDSCs suppression by Wnt signaling, its presence in the Wnt active tumor microenvironment is highly contradictory. This was partially explained with the presence of Dkk1 secreted by the tumor stroma, which inhibits Beta-catenin in MDSCs (90). To understand this puzzle of interplay between Wnt signaling and MDSCs, further study is needed that can correlate various driving factors working in different context. Wnt signaling shows a negative correlation with TAA presentation. Tumor-induced β-catenin signaling functions in DCs to execute an exhaustive immune-effector phenotype in the infiltrating antitumor CTLs (91). Wnt3a regulates canonical β-catenin signaling in DCs, whereas the non-canonical signaling cascade is regulated by Wnt5a, leading to a tolerogenic DC phenotype (92, 93). Canonical Wnt signaling can potentially regulate DC activation and maturation (94). It has been shown that β-catenin deletion in DCs increased the surface expression of co-stimulatory markers CD80 and CD86 and decreased surface expression of co-inhibitory molecules like PDL1 and PDL2 (95). This phenotype of DC exhibits a positive correlation with antitumor immunity. On the contrary, β-catenin-active tumors do not react to anti-CTLA-4/anti-PD-1 immunotherapy (96). Therefore, a combinatorial therapy of the Wnt inhibitor specifically targeting DCs along with PD1 and CTLA4 immune therapy can work better for patients not responding to cancer immune therapy.

## Wnt-β-Catenin Pathway: A Target for Therapeutic Interventions in Cancer

Apart from playing a vital role in various cellular activities like organogenesis and stem cell regeneration, the Wnt/β-catenin pathway is also associated with cancers, such as colorectal, cervical, breast, lung, oral squamous cell carcinoma, and hematopoietic malignancies and their recurrence. In this context, targeting aberrant Wnt/β-catenin signaling as a therapeutic intervention to combat the abovementioned cancers seems a lucrative approach. However, the challenge here lies in identifying effective agents that can target the Wnt pathway without tampering the normal cellular functions like tissue repair and homeostasis, the renewal of stem cells, and survival. The detailed action of Wnt signaling in both canonical and non-canonical pathway has been summarized in the prior sections.

Wnt pathway is observed to be upregulated in cancers. Activation of Wnt leads to the loss of function of APC, which is a negative regulator of cell proliferation. Inhibitors of the Wnt signaling pathway can therefore have therapeutic values in cancer treatment, and multiple such targets have been identified wherein inhibitors act at different steps of Wnt signaling pathway. Broadly, these inhibitors can be classified into two categories: 1. Inhibitors of Wnt-receptor complex, and 2. β-catenin destruction complex inhibitors. The detailed mechanism of action of these inhibitors and their subtypes has been briefed below. Also, a list summarizing such drugs and their clinical trial status has been appended in Table 2.

**Table 2:** List of drugs for specific diseases under clinical trials and their targets.

**S. no**.	**Name**	**Company**	**Target**	**Disease**	**Clinical phase**
1.	OMP18R5 (vantictumab)	OncoMed Pharmaceuticals	frizzled	Solid tumors	Phase I (dose escalation study)
2.	OMP-54F28	OncoMed pharmaceuticals/bayer	Wnt	Solid tumors	Phase I
3.	LGK974	Novartis pharmaceuticals	Porcupine	Melanoma, breast cancer, and pancreatic adenocarcinoma	Phase I
4.	CWP232291	JW pharmaceutical	β-catenin	Acute myeloid leukemia	Phase I
5.	PRI-724	Prism/Eisai pharmaceuticals	β-catenin/CBP	Advanced myeloid malignancies	Phase I (dose escalation study)
6.	IWR1	Tocris bioscience	Tankyrases 1, 2 inhibitor	Osteosarcoma	Preclinical
7.	XAV939	Novartis	Tankyrases 1, 2 inhibitor	Neuroblastoma	Preclinical
8.	NSC668036	Tocris bioscience	Disheveled	Fibrotic lung disease	Preclinical
9.	ICG-001	Prism pharma	CREB binding protein/CBP	Acute myeloid leukemia Chronic myeloid leukemia	Phase I Phase II
10.	DKN-01	Leap therapeutics	DKK, dickkopf-related protein	Multiple Myeloma	Phase I, II

### Inhibitors of the Wnt-Receptor Complex

#### Porcupine Inhibitors

Porcupine, abbreviated as PORCN, is an O-acyltransferase (MBOAT) and plays a crucial role in Wnt ligand secretion by providing the Wnt proteins with a palmitoyl group. It recently became a highly druggable target for inhibiting Wnt signaling pathways (97, 98). Moreover, PORCN is the only enzyme specific to the Wnt cascade that is found to be upregulated in mouse cancer models, and it is often regarded as a poor prognosis marker for head and neck squamous cell cancers (99). *In-vivo* studies have shown that selectively inhibiting PORCN by using LGK974 blocks Wnt signaling and subsequently tumor growth (100). Independent study has shown effectiveness of LGK974 *in-vitro* on head and neck cancer cells with NOTCH1 mutations. This molecule is now undergoing phase 1 and phase 2 clinical trials. Another promising molecule for PORCN inhibition is an oral, selective small molecule inhibitor, ETC-159, of porcupine, which has shown favorable results in preclinical studies and has now entered phase 1 of clinical trials (99).

#### Antibodies Against Wnt Family Proteins

Several tumors have been marked to be overexpressing Wnt ligands and/or their receptors. This specific interaction is also an attractive target, and many groups are working on antibodies that can act at this point and lead to the inhibition of further downstream signaling. Monoclonal antibodies (MAbs) designed to bind Wnt1 and Wnt2 have shown to lead to tumor suppression in a plethora of malignancies, including, but not limited to, melanoma, colorectal cancers, and non-small cell lung carcinoma (101).

OncoMed Pharmaceuticals/Bayer manufactured a MAb that can target 5 Frizzled receptors and named it as OMP-18R5 or Vantictumab (101). Its safety and efficacy in many cancers are being evaluated alone or combined with other chemotherapeutic regimes. A phase Ib study of a combination of OMP-18R5 with other drugs was carried out in patients with stage IV pancreatic adenocarcinoma and metastatic HER2-negative breast cancer (102, 103). Another recombinant fusion protein that blocks the Wnt signaling is OMP-54F28 (or Ipafricept). A first-in-human phase I study of this decoy receptor for Wnt ligands is presently being carried out in patients with advanced stages of solid tumors (104). It binds to the Wnt ligand through the adomain present in the extracellular part of the human Frizzled 8 receptor (fused to a human IgG1 Fc fragment). This also binds to Wnt ligands and blocks the downstream signaling. In xenograft models of ovarian cancer, Ipafricept has shown a reduction in the frequency of stem cells, suppressed tumor formation, and stimulate differentiation. Also in a combinatorial approach, treatment with OMP-54F28 prior to taxane chemotherapy displays synergy and, therefore, superior antitumor efficacy (105).

### β-Catenin-Destruction Complex Inhibitors

#### Tankyrase or PARP5 Inhibitors

The Wnt/β-catenin pathway is associated with the PARP [Poly (ADP-ribose) polymerases] family of proteins. Of the PARP family, two isoforms, namely PARP5a (Tankyrase 1) and PARP5b (Tankyrase 2), are known to degrade the axin by the ubiquitin–proteasome dependent pathway (106). Inhibitors of these isoforms, XAV939 and IWR-1, help in maintaining the optimum Axin levels. Another inhibitor- NVP-TNKS656 used in murine xenografts models and colorectal cancer patient-derived sphere culture studies showed a high β-catenin level even in the presence of AKT and PI3K inhibitors signifying that the tankyrase inhibitor could overcome resistance to AKT and PI3K inhibitors. It was also associated with high FOXO3A (Forkhead box O3) activity. The setback of this study lies in the trepidations of gastrointestinal toxicity associated with these inhibitors, and further studies are needed to verify their safety and efficacy. Selective tankyrase inhibitors, MN-64 and CMP8, with the capacity to bind to the nicotinamide subsite of tankyrases are amongst the best inhibitors and have demonstrated nanomolar potencies (107).

#### Disheveled Inhibitors

Disheveled binds to the frizzled receptor on its C-terminal region via its PDZ domain. This Frizzled–disheveled interaction is a target for some notable inhibitors like NSC668036, FJ9, and 3289–8625, leading to inhibition of the Wnt signal transduction pathway and thereby causing cancer regression (108). Sulindac is a FDA approved disheveled inhibitor which can inhibit the proliferation of lung cancer A549 cells (108, 109).

#### Inhibitors of Transcription Complex

Wnt signaling involves a plethora of intermediate steps, and there is therefore a constantly evolving pursuit to find agents that can target the downstream steps of this pathway. A high-throughput ELISA-based screening was carried out to shortlist small molecules that could possibly target the interaction between β-catenin and the transcription factor TCF4. Eight inhibitors were identified to be effective in perturbing the β-catenin/TCF complex in a dose-dependent manner. LF3, a 4-thioureido-benzenesulfonamide derivative, possesses the capacity to perturb this interaction in colon cancer (110). However, this interaction is not very specific, causing many off-target effects.

#### Antagonists of Wnt Co-activator

The prerequisite step for Wnt activation is the interaction of β-catenin with its transcriptional co-activator CBP. Small molecule antagonists that have the capacity to inhibit this interaction can serve as a potential therapeutic agent. CBPPRI-724 is a first-in-class antagonist that acts in line with this approach. This molecule has shown promising results in the preclinical stage with pancreatic cancer cells. It has been proven to promote differentiation of CSCs, inhibition of stroma formation, and decrease the metastatic potential of cancer cells (111).

#### Wnt5a Mimetics

Wnt5a acts as a tumor suppressor in various cancers, and its downregulation is often associated with lower disease-free survival in primary breast cancers and also in hematopoietic, prostate, and colon cancers. Foxy-5 is a hexapeptide mimic of Wnt5a which is synthesized to possess Wnt5a-like properties that can impair cancer cell migration. After a successful phase 1 study in colon/breast/prostate cancer patients showing no toxicity, phase 1b trials are ongoing and expected to show promising therapeutic value (112). Also, peptides derived by modifying the Wnt5a ligand sequence have shown the capability to mimic the Wnt5a molecule. It can bind to the Fzd-5 receptor in a human breast tumor cell line and impair metastatic ability (113).

#### Gamma Secretase Inhibitor

Wnt signaling has now been shown to be closely associated with other pathways, like the Notch signaling pathway, and Notch1 is thought to be acting as a connecting link between them. Treatment of CSCs with GSI agents leads to the induction of apoptosis and inhibition of tumor sphere formation of CD44^+^ CSCs. MK-0752 in combination with ridaforolimus (MK-8669) is currently in phase I trial of patients with solid tumors (114). MK-0752 with docetaxel has entered phase II trials in breast cancer patients (115). Another selective GSI, PF-03084014, showed a reduction in tumor cell migration and mammosphere formation *in vitro*, and a marked decrease in tumor cell self-renewal ability *in vivo* (116). The phase I trials in triple negative breast cancer patients in combination with docetaxel, however, showed gastrointestinal toxicity.

#### Hedgehog Inhibitors

Another pathway involved in crosstalk with the Wnt pathway is the sonic hedgehog pathway with sFRP-1 being the mediator between them. FDA-approved SMO (Smoothened) inhibitor- Vismodegib binds directly to SMO and inhibits the progression of advanced basal cell cancers (117). It is also being studied in the context of other cancers, like gastric and prostate cancer, and is currently undergoing phase I and II trials. Another FDA-approved SMO antagonist is NVP-LDE225 (Erismodegib or sonidegib), used for advanced cases of basal cell carcinomas (118). Erismodegib is also being studied in phase I and II clinical trials for other cancer types.

Apart from different chemical inhibitors, many natural compounds have shown to target the Wnt pathway directly or indirectly. For example Indole-3-carbinol (I3C), a natural compound present in broccoli can inhibit WWS1-mediated proteasomal degradation of PTEN, which has a direct interaction with the Wnt/beta-catenin pathway, leading to tumor regression both *in vitro* and *in vivo* (119, 120). Also, there are various repressor proteins reported to downregulate β-catenin. One such protein is SMAR1, a tumor suppressor known to be downregulated in higher grades of cancer and that inhibits β-catenin transcription (121, 122). It inhibits β-catenin transcription as well as negatively regulate mir371-373, which is known to target DKK1—an inhibitor of Wnt signaling (121, 123, 124). Aberrant Wnt signaling or CDC20 mediated degradation, downregulate SMAR1 expression thereby promoting tumorigenesis and cancer progression (125). Compounds such as I3C (119) and putative SMAR1 stabilizing compounds can be potential therapeutic targets to inhibit Wnt/β-catenin pathway in different types of cancer cells, thereby regressing tumors.

**Figure 4:**
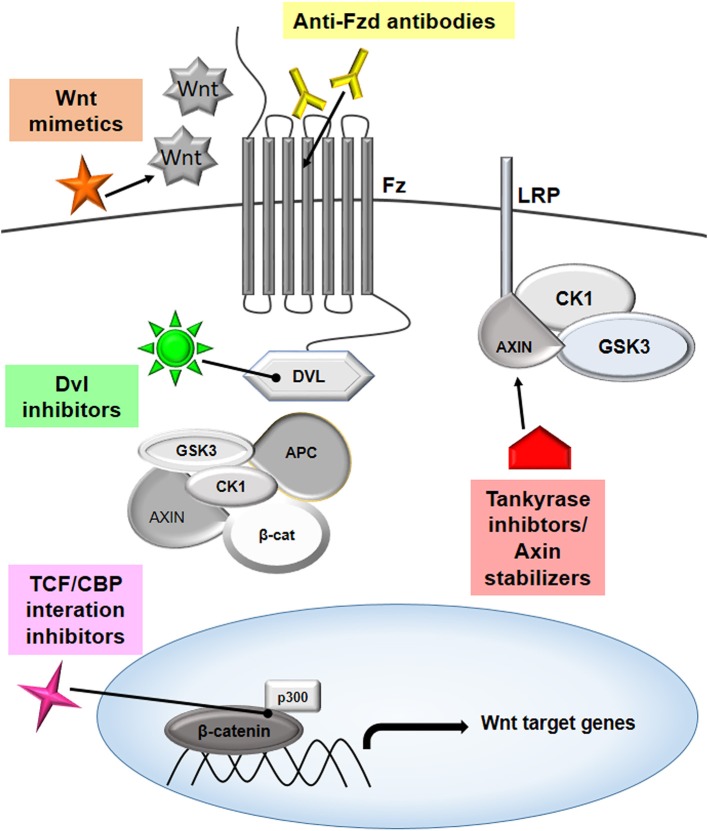
Targeting Wnt signaling to combat cancer. The Wnt pathway has multiple players that can be effectively targeted to modulate the signaling cascade, thereby inhibiting cancer proliferation. The targets that are showing promising results are highlighted in the figure. Several therapeutic molecules, such as anti-Frizzled antibodies, mimetics of Wnt molecules, disheveled inhibitors, tankyrase inhibitors, axin stabilizers, and inhibitors of TCF/CBP interactions are being evaluated in different phases of clinical studies.

To conclude, Wnt signaling can be targeted at various steps to develop potential therapy against cancer, and this is summarized in Figure 4.

#### Challenges of Wnt Inhibition-Based Therapies

Inhibiting cancer by targeting the Wnt signaling pathway has been a hotspot for the last four decades. Various molecules involved in this pathway have been studied in depth and proposed as innovative targets for anti-tumor therapy, and some have also found uses in the treatment of neurodegenerative disease, such as Parkinson's disease. Despite showing promising results, no such drugs have been approved for clinical use. This may be attributed to the fact that Wnt/β catenin signaling is crucial for stem cell pool maintenance and also in the regeneration of tissues and organs. Thus, tweaking this pathway can also affect the normal Wnt-dependent activities. Some of the drugs targeting Wnt signaling have shown dose-limiting gastrointestinal toxicity and upregulation of markers for bone formation and growth. Apart from direct roles, Wnt signaling is also known to cross functions with other pathways that are involved in cell signaling. A better understanding of these crosstalks and the development of combination therapy that can target specific molecules without disrupting normal cellular functions or using natural compounds may be the choice of future research.

## Concluding Remarks and Future Directions

Tumor cells require a milieu of growth factors, secretory molecules, and crosstalk between different cells present in the tumor microenvironment for their growth and proliferation. An increasing amount of evidence suggests that Wnt signaling acts as bridge between tumor cells and the tumor microenvironment for their preferential growth and progression. Stromal cells and inflammatory cells present in the extracellular matrix of the tumor microenvironment secrete Wnt ligands that promote tumor invasion, metastasis, and tolerance. Although Wnt signaling is important for hematopoiesis and immune cell development and proliferation, the larger picture suggest that a context-dependent regulation of Wnt signaling in the tumor microenvironment renders immune cells tolerance toward immune escape by inhibiting tumor antigen presentation. Various reports unanimously support the hypothesis that both the canonical and non-canonical Wnt pathway in the tumor microenvironment induce a signaling cascade, leading to EMT, metastasis, and cancer stem cell maintenance. With the advent of new high throughput technologies, we are in a better state of understanding of the mechanism of both the canonical and non-canonical Wnt signaling pathway. This understanding is the basis of translational research targeting the important molecules of this pathway and their downstream effectors in cancer and leveraging this knowledge toward designing personalized medicine. Several Wnt/β-catenin inhibitors that have the potential for use in anticancer therapies have been recognized. Clinical trials for these drugs are in various stages, and some of them are showing immense potential for future anticancer therapy. Further, these inhibitors, in combination with existing immune therapy or using natural compounds, can do wonders in eliminating higher grades of cancer and metastatic tumors. However, there remains a scope for further study to investigate and counter the side effects of harnessing the Wnt/β-catenin signaling, as it is important for cellular homeostasis.

## Author Contributions

Conception of idea was done by SC, AA, and SP. Manuscript writing and editing was done by all the authors.

### Conflict of Interest

The authors declare that the research was conducted in the absence of any commercial or financial relationships that could be construed as a potential conflict of interest.

## References

[1] Casey SC Vaccari M Al-Mulla F Al-Temaimi R Amedei A Barcellos-Hoff MH. The effect of environmental chemicals on the tumor microenvironment. Carcinogenesis. (2015) 36:S160–83. 10.1093/carcin/bgv035 26106136 PMC4565612

[2] Gattinoni L Ji Y Restifo NP. Wnt/β- catenin signaling in T-cell immunity and cancer immunotherapy. Clin Cancer Res. (2010) 16:4695–701. 10.1158/1078-0432.CCR-10-0356 20688898 PMC3393131

[3] Zhan T Rindtorff N Boutros M. Wnt signaling in cancer. Oncogene. (2017) 36:1461–73. 10.1038/onc.2016.304 27617575 PMC5357762

[4] de Sousa E Melo F Vermeulen L Wnt signaling in cancer stem cell biology. Cancers. (2016) 8:E60 10.3390/cancers8070060 27355964 PMC4963802

[5] Suryawanshi A Tadagavadi RK Swafford D Manicassamy S. Modulation of inflammatory responses by Wnt/β-catenin signaling in dendritic cells: a novel immunotherapy target for autoimmunity and cancer. Front Immunol. (2016) 7:460. 10.3389/fimmu.2016.00460 27833613 PMC5081350

[6] Endo M Nishita M Fujii M Minami Y. Insight into the role of Wnt5a-induced signaling in normal and cancer cells. Int Rev Cell Mol Biol. (2015) 314:117–48. 10.1016/bs.ircmb.2014.10.003 25619716

[7] Staal FJT Luis TC Tiemessen MM. WNT signalling in the immune system: WNT is spreading its wings. Nat Rev Immunol. (2008) 8:581–93. 10.1038/nri2360 18617885

[8] Swafford D Manicassamy S. Wnt signaling in dendritic cells: its role in regulation of immunity and tolerance. Discov Med. (2015) 19:303–10. 25977193 PMC4513356

[9] Jenny FH Basler K. Powerful Drosophila screens that paved the wingless pathway. Fly. (2014) 8:218–25. 10.4161/19336934.2014.985988 25565425 PMC4594357

[10] Nüsslein-Volhard C Wieschaus E. Mutations affecting segment number and polarity in Drosophila. Nature. (1980) 287:795–801. 10.1038/287795a0 6776413

[11] Rijsewijk F Schuermann M Wagenaar E Parren P Weigel D Nusse R. The Drosophila homolog of the mouse mammary oncogene int-1 is identical to the segment polarity gene wingless. Cell. (1987) 50:649–57. 10.1016/0092-8674(87)90038-9 3111720

[12] van Amerongen R Nusse R. Towards an integrated view of Wnt signaling in development. Development. (2009) 136:3205–14. 10.1242/dev.033910 19736321

[13] Kikuchi A Yamamoto H Sato A Matsumoto S. New insights into the mechanism of Wnt signaling pathway activation. Int Rev Cell Mol Biol. (2011) 291:21–71. 10.1016/B978-0-12-386035-4.00002-1 22017973

[14] MacDonald BT Tamai K He X. Wnt/β-catenin signaling: components, mechanisms, and diseases. Dev Cell. (2009) 17:9. 10.1016/j.devcel.2009.06.016 19619488 PMC2861485

[15] Clevers H. Wnt/beta-catenin signaling in development and disease. Cell. (2006) 127:469–80. 10.1016/j.cell.2006.10.018 17081971

[16] Niehrs C. The complex world of WNT receptor signalling. Nat Rev Mol Cell Biol. (2012) 13:767–79. 10.1038/nrm3470 23151663

[17] Wong GT Gavin BJ McMahon AP. Differential transformation of mammary epithelial cells by Wnt genes. Mol Cell Biol. (1994) 14:6278–86. 10.1128/MCB.14.9.6278 8065359 PMC359154

[18] Tamai K Zeng X Liu C Zhang X Harada Y Chang Z. A mechanism for Wnt coreceptor activation. Mol Cell. (2004) 13:149–56. 10.1016/S1097-2765(03)00484-2 14731402

[19] Mao J Wang J Liu B Pan W Farr GH Flynn C. Low-density lipoprotein receptor-related protein-5 binds to Axin and regulates the canonical Wnt signaling pathway. Mol Cell. (2001) 7:801–9. 10.1016/S1097-2765(01)00224-6 11336703

[20] Davidson G Wu W Shen J Bilic J Fenger U Stannek P. Casein kinase 1 γ couples Wnt receptor activation to cytoplasmic signal transduction. Nature. (2005) 438:867–72. 10.1038/nature04170 16341016

[21] Krishnamurthy N Kurzrock R. Targeting the Wnt/beta-catenin pathway in cancer: update on effectors and inhibitors. Cancer Treat Rev. (2018) 62:50–60. 10.1016/j.ctrv.2017.11.002 29169144 PMC5745276

[22] Levanon D Goldstein RE Bernstein Y Tang H Goldenberg D Stifani S. Transcriptional repression by AML1 and LEF-1 is mediated by the TLE/Groucho corepressors. Proc Natl Acad Sci USA. (1998) 95:11590–5. 10.1073/pnas.95.20.11590 9751710 PMC21685

[23] Brantjes H Roose J van De Wetering M Clevers H. All Tcf HMG box transcription factors interact with Groucho-related co-repressors. Nucleic Acids Res. (2001) 29:1410–9. 10.1093/nar/29.7.1410 11266540 PMC31284

[24] Gordon MD Nusse R. Wnt signaling: multiple pathways, multiple receptors, and multiple transcription factors. J Biol Chem. (2006) 281:22429–33. 10.1074/jbc.R600015200 16793760

[25] Katoh M. Canonical and non-canonical WNT signaling in cancer stem cells and their niches: cellular heterogeneity, omics reprogramming, targeted therapy and tumor plasticity (Review). Int J Oncol. (2017) 51:1357–69. 10.3892/ijo.2017.4129 29048660 PMC5642388

[26] Katoh M. WNT/PCP signaling pathway and human cancer (Review). Oncol Rep. (2005) 14:1583–8. 10.3892/or.14.6.1583 16273260

[27] Tao W Pennica D Xu L Kalejta RF Levine AJ. Wrch-1, a novel member of the Rho gene family that is regulated by Wnt-1. Genes Dev. (2001) 15:1796–807. 10.1101/gad.894301 11459829 PMC312736

[28] Boutros M Paricio N Strutt DI Mlodzik M. Dishevelled activates JNK and discriminates between JNK pathways in planar polarity and wingless signaling. Cell. (1998) 94:109–18. 10.1016/S0092-8674(00)81226-X 9674432

[29] Ishitani T Kishida S Hyodo-Miura J Ueno N Yasuda J Waterman M. The TAK1-NLK mitogen-activated protein kinase cascade functions in the Wnt-5a/Ca(2+) pathway to antagonize Wnt/beta-catenin signaling. Mol Cell Biol. (2003) 23:131–9. 10.1128/MCB.23.1.131-139.2003 12482967 PMC140665

[30] Dejmek J Safholm A Kamp Nielsen C Andersson T Leandersson K Wnt-5a/Ca2+-induced NFAT activity is counteracted by Wnt-5a/Yes-Cdc42-Casein Kinase 1 signaling in human mammary epithelial cells. Mol Cell Biol. (2006) 26:6024–36. 10.1128/MCB.02354-05 16880514 PMC1592795

[31] De A. Wnt/Ca2+ signaling pathway: a brief overview. Acta Biochim Biophys Sin. (2011) 43:745–56. 10.1093/abbs/gmr079 21903638

[32] Quail DF Joyce JA. Microenvironmental regulation of tumor progression and metastasis. Nat Med. (2013) 19:1423–37. 10.1038/nm.3394 24202395 PMC3954707

[33] Joyce JA Pollard JW. Microenvironmental regulation of metastasis. Nat Rev Cancer. (2009) 9:239–52. 10.1038/nrc2618 19279573 PMC3251309

[34] Egeblad M Nakasone ES Werb Z. Tumors as organs: complex tissues that interface with the entire organism. Dev Cell. (2010) 18:884–901. 10.1016/j.devcel.2010.05.012 20627072 PMC2905377

[35] Alfaro C Sanmamed MF Rodríguez-Ruiz ME Teijeira Á Oñate C González Á. Interleukin-8 in cancer pathogenesis, treatment and follow-up. Cancer Treat Rev. (2017) 60:24–31. 10.1016/j.ctrv.2017.08.004 28866366

[36] Gonzalez-Aparicio M Alfaro C. Influence of Interleukin-8 and neutrophil extracellular trap (NET) formation in the tumor microenvironment: is there a pathogenic role? J Immunol Res. (2019) 2019:6252138. 10.1155/2019/6252138 31093511 PMC6481028

[37] Waugh DJJ Wilson C. The interleukin-8 pathway in cancer. Clin Cancer Res. (2008) 14:6735–41. 10.1158/1078-0432.CCR-07-4843 18980965

[38] Wu T Dai Y. Tumor microenvironment and therapeutic response. Cancer Lett. (2017) 387:61–8. 10.1016/j.canlet.2016.01.043 26845449

[39] Klemm F Joyce JA. Microenvironmental regulation of therapeutic response in cancer. Trends Cell Biol. (2015) 25:198–213. 10.1016/j.tcb.2014.11.006 25540894 PMC5424264

[40] Wang M Zhao J Zhang L Wei F Lian Y Wu Y. Role of tumor microenvironment in tumorigenesis. J Cancer. (2017) 8:761–73. 10.7150/jca.17648 28382138 PMC5381164

[41] Östman A Augsten M. Cancer-associated fibroblasts and tumor growth – bystanders turning into key players. Curr Opin Genet Dev. (2009) 19:67–73. 10.1016/j.gde.2009.01.003 19211240

[42] Paraiso KHT Smalley KSM. Fibroblast-mediated drug resistance in cancer. Biochem Pharmacol. (2013) 85:1033–41. 10.1016/j.bcp.2013.01.018 23376122

[43] Gabrilovich DI Ostrand-Rosenberg S Bronte V. Coordinated regulation of myeloid cells by tumours. Nat Rev Immunol. (2012) 12:253–68. 10.1038/nri3175 22437938 PMC3587148

[44] Balkwill F Mantovani A. Inflammation and cancer: back to Virchow? Lancet. (2001) 357:539–45. 10.1016/S0140-6736(00)04046-0 11229684

[45] Jiang X Wang J Deng X Xiong F Ge J Xiang B. Role of the tumor microenvironment in PD-L1/PD-1-mediated tumor immune escape. Mol Cancer. (2019) 18:1–17. 10.1186/s12943-018-0928-4 30646912 PMC6332843

[46] Kim S-H Turnbull J Guimond S. Extracellular matrix and cell signalling: the dynamic cooperation of integrin, proteoglycan and growth factor receptor. J Endocrinol. (2011) 209:139–51. 10.1530/JOE-10-0377 21307119

[47] Netti PA Berk DA Swartz MA Grodzinsky AJ Jain RK. Role of extracellular matrix assembly in interstitial transport in solid tumors. Cancer Res. (2000) 60:2497–503. 10811131

[48] De Sanctis F Ugel S Facciponte J Facciabene A. The dark side of tumor-associated endothelial cells. Semin Immunol. (2018) 35:35–47. 10.1016/j.smim.2018.02.002 29490888

[49] Talmadge JE Gabrilovich DI. History of myeloid-derived suppressor cells. Nat Rev Cancer. (2013) 13:739–52. 10.1038/nrc3581 24060865 PMC4358792

[50] Mulligan JK Day TA Gillespie MB Rosenzweig SA Young MRI. Secretion of vascular endothelial growth factor by oral squamous cell carcinoma cells skews endothelial cells to suppress T-cell functions. Hum Immunol. (2009) 70:375–82. 10.1016/j.humimm.2009.01.014 19480853 PMC2746465

[51] Duong MN Geneste A Fallone F Li X Dumontet C Muller C. The fat and the bad: mature adipocytes, key actors in tumor progression and resistance. Oncotarget. (2017) 8:57622–41. 10.18632/oncotarget.18038 28915700 PMC5593672

[52] Corti A. Chromogranin A and the tumor microenvironment. Cell Mol Neurobiol. (2010) 30:1163–70. 10.1007/s10571-010-9587-8 21080056 PMC11498746

[53] Thiery JP Sleeman JP. Complex networks orchestrate epithelial–mesenchymal transitions. Nat Rev Mol Cell Biol. (2006) 7:131–42. 10.1038/nrm1835 16493418

[54] He X. Unwinding a path to nuclear β-catenin. Cell. (2006) 127:40–2. 10.1016/j.cell.2006.09.016 17018273

[55] Jing Y Han Z Zhang S Liu Y Wei L. Epithelial-mesenchymal transition in tumor microenvironment. Cell Biosci. (2011) 1:29. 10.1186/2045-3701-1-29 21880137 PMC3179439

[56] Fodde R Brabletz T. Wnt/β-catenin signaling in cancer stemness and malignant behavior. Curr Opin Cell Biol. (2007) 19:150–8. 10.1016/j.ceb.2007.02.007 17306971

[57] Baykal C Ayhan A Al A Yüce K Ayhan A. Overexpression of the c-Met/HGF receptor and its prognostic significance in uterine cervix carcinomas. Gynecol Oncol. (2003) 88:123–9. 10.1016/S0090-8258(02)00073-2 12586590

[58] Rasola A Fassetta M De Bacco F D'Alessandro L Gramaglia D Di Renzo MF. A positive feedback loop between hepatocyte growth factor receptor and β-catenin sustains colorectal cancer cell invasive growth. Oncogene. (2007) 26:1078–87. 10.1038/sj.onc.1209859 16953230

[59] Yang L Lin C Liu Z-R. P68 RNA Helicase mediates PDGF-induced epithelial mesenchymal transition by displacing Axin from β-Catenin. Cell. (2006) 127:139–55. 10.1016/j.cell.2006.08.036 17018282

[60] Des Guetz G Uzzan B Nicolas P Cucherat M Morere J-F Benamouzig R. Microvessel density and VEGF expression are prognostic factors in colorectal cancer. Meta-analysis of the literature. Br J Cancer. (2006) 94:1823–32. 10.1038/sj.bjc.6603176 16773076 PMC2361355

[61] Sales CBS Buim MEC de Souza RO de Faro Valverde L Mathias Machado MC Reis MG. Elevated VEGFA mRNA levels in oral squamous cell carcinomas and tumor margins: a preliminary study. J Oral Pathol Med. (2016) 45:481–5. 10.1111/jop.12398 26861159

[62] Birdsey GM Shah AV Dufton N Reynolds LE Osuna Almagro L Yang Y. The endothelial transcription factor ERG promotes vascular stability and growth through Wnt/β-catenin signaling. Dev Cell. (2015) 32:82–96. 10.1016/j.devcel.2014.11.016 25584796 PMC4292982

[63] Takahashi H Sakakura K Kudo T Toyoda M Kaira K Oyama T. Cancer-associated fibroblasts promote an immunosuppressive microenvironment through the induction and accumulation of protumoral macrophages. Oncotarget. (2017) 8:8633–47. 10.18632/oncotarget.14374 28052009 PMC5352428

[64] Kramer N Schmöllerl J Unger C Nivarthi H Rudisch A Unterleuthner D. Autocrine WNT2 signaling in fibroblasts promotes colorectal cancer progression. Oncogene. (2017) 36:5460–72. 10.1038/onc.2017.144 28553956

[65] Castellone MD Teramoto H Williams BO Druey KM Gutkind JS. Prostaglandin E2 promotes colon cancer cell growth through a Gs-Axin- -catenin signaling axis. Science. (2005) 310:1504–10. 10.1126/science.1116221 16293724

[66] Smith K Bui TD Poulsom R Kaklamanis L Williams G Harris AL. Up-regulation of macrophage wnt gene expression in adenoma-carcinoma progression of human colorectal cancer. Br J Cancer. (1999) 81:496–502. 10.1038/sj.bjc.6690721 10507776 PMC2362915

[67] Ojalvo LS Whittaker CA Condeelis JS Pollard JW. Gene expression analysis of macrophages that facilitate tumor invasion supports a role for Wnt-signaling in mediating their activity in primary mammary tumors. J Immunol. (2010) 184:702–12. 10.4049/jimmunol.0902360 20018620 PMC3226722

[68] Rao S Lobov IB Vallance JE Tsujikawa K Shiojima I Akunuru S. Obligatory participation of macrophages in an angiopoietin 2-mediated cell death switch. Development. (2007) 134:4449–58. 10.1242/dev.012187 18039971 PMC3675770

[69] Yang Y Ye Y-C Chen Y Zhao J-L Gao C-C Han H. Crosstalk between hepatic tumor cells and macrophages via Wnt/β-catenin signaling promotes M2-like macrophage polarization and reinforces tumor malignant behaviors. Cell Death Dis. (2018) 9:793. 10.1038/s41419-018-0818-0 30022048 PMC6052107

[70] Kaler P Augenlicht L Klampfer L. Macrophage-derived IL-1β stimulates Wnt signaling and growth of colon cancer cells: a crosstalk interrupted by vitamin D3. Oncogene. (2009) 28:3892–902. 10.1038/onc.2009.247 19701245 PMC2783659

[71] Gregorieff A Pinto D Begthel H Destrée O Kielman M Clevers H. Expression pattern of Wnt signaling components in the adult intestine. Gastroenterology. (2005) 129:626–38. 10.1016/j.gastro.2005.06.007 16083717

[72] Ormestad M Astorga J Landgren H Wang T Johansson BR Miura N. Foxf1 and Foxf2 control murine gut development by limiting mesenchymal Wnt signaling and promoting extracellular matrix production. Development. (2006) 133:833–43. 10.1242/dev.02252 16439479

[73] Austin TW Solar GP Ziegler FC Liem L Matthews W. A role for the Wnt gene family in hematopoiesis: expansion of multilineage progenitor cells. Blood. (1997) 89:3624–35. 10.1182/blood.V89.10.3624.3624_3624_3635 9160667

[74] Cheng X Huber TL Chen VC Gadue P Keller GM. Numb mediates the interaction between Wnt and Notch to modulate primitive erythropoietic specification from the hemangioblast. Development. (2008) 135:3447–58. 10.1242/dev.025916 18799543 PMC3039875

[75] Clements WK Kim AD Ong KG Moore JC Lawson ND Traver D. A somitic Wnt16/Notch pathway specifies haematopoietic stem cells. Nature. (2011) 474:220–4. 10.1038/nature10107 21654806 PMC3304471

[76] Reya T Duncan AW Ailles L Domen J Scherer DC Willert K. A role for Wnt signalling in self-renewal of haematopoietic stem cells. Nature. (2003) 423:409–14. 10.1038/nature01593 12717450

[77] Trowbridge JJ Xenocostas A Moon RT Bhatia M. Glycogen synthase kinase-3 is an *in vivo* regulator of hematopoietic stem cell repopulation. Nat Med. (2006) 12:89–98. 10.1038/nm1339 16341242

[78] Rothenberg EV Moore JE Yui MA. Launching the T-cell-lineage developmental programme. Nat Rev Immunol. (2008) 8:9–21. 10.1038/nri2232 18097446 PMC3131407

[79] Osada M Jardine L Misir R Andl T Millar SE Pezzano M. DKK1 Mediated inhibition of Wnt signaling in postnatal mice leads to loss of TEC progenitors and thymic degeneration. PLoS ONE. (2010) 5:e9062. 10.1371/journal.pone.0009062 20161711 PMC2817005

[80] Reya T O'Riordan M Okamura R Devaney E Willert K Nusse R. Wnt signaling regulates B lymphocyte proliferation through a LEF-1 dependent mechanism. Immunity. (2000) 13:15–24. 10.1016/S1074-7613(00)00004-2 10933391

[81] Osugui L de Roo JJ de Oliveira VC Sodré ACP Staal FJT Popi AF. B-1 cells and B-1 cell precursors prompt different responses to Wnt signaling. PLoS ONE. (2018) 13:e0199332. 10.1371/journal.pone.0199332 29928002 PMC6013157

[82] Marcus A Gowen BG Thompson TW Iannello A Ardolino M Deng W. Recognition of tumors by the innate immune system and natural killer cells. Adv Immunol. (2014) 122:91–128. 10.1016/B978-0-12-800267-4.00003-1 24507156 PMC4228931

[83] Escors D. Tumour immunogenicity, antigen presentation, and immunological barriers in cancer immunotherapy. New J Sci. (2014) 2014:1–25. 10.1155/2014/734515 24634791 PMC3952940

[84] Marvel D Gabrilovich DI. Myeloid-derived suppressor cells in the tumor microenvironment: expect the unexpected. J Clin Invest. (2015) 125:3356–64. 10.1172/JCI80005 26168215 PMC4588239

[85] Lanitis E Dangaj D Irving M Coukos G. Mechanisms regulating T-cell infiltration and activity in solid tumors. Ann Oncol. (2017) 28:xii18–xii32. 10.1093/annonc/mdx238 29045511

[86] Capietto AH Kim S Sanford DE Linehan DC Hikida M Kumosaki T. Down-regulation of PLCγ2-β-catenin pathway promotes activation and expansion of myeloid-derived suppressor cells in cancer. J Exp Med. (2013) 210:2257–71. 10.1084/jem.20130281 24127488 PMC3804931

[87] Olsen JJ Pohl SÖ Deshmukh A Visweswaran M Ward NC Arfuso F. The role of Wnt signalling in angiogenesis. Clin Biochem Rev. (2017) 38:131–42. 29332977 PMC5759160

[88] Valencia J Martínez VG Hidalgo L Hernández-López C Canseco NM Vicente Á. Wnt5a signaling increases IL-12 secretion by human dendritic cells and enhances IFN-γ production by CD4+ T cells. Immunol Lett. (2015) 162:188–99. 10.1016/j.imlet.2014.08.015 25196330

[89] Steding CE Wu ST Zhang Y Jeng MH Elzey BD Kao C. The role of interleukin-12 on modulating myeloid-derived suppressor cells, increasing overall survival and reducing metastasis. Immunology. (2011) 133:221–38. 10.1111/j.1365-2567.2011.03429.x 21453419 PMC3088984

[90] D'Amico L Mahajan S Capietto AH Yang Z Zamani A Ricci B. Dickkopf-related protein 1 (Dkk1) regulates the accumulation and function of myeloid derived suppressor cells in cancer. J Exp Med. (2016) 213:827–40. 10.1084/jem.20150950 27045006 PMC4854727

[91] Luke JJ Bao R Sweis RF Spranger S Gajewski TF. WNT/β-catenin pathway activation correlates with immune exclusion across human cancers. Clin Cancer Res. (2019) 25:3074–83. 10.1158/1078-0432.CCR-18-1942 30635339 PMC6522301

[92] Valencia J Hernández-López C Martínez VG Hidalgo L Zapata AG Vicente Á. Wnt5a skews dendritic cell differentiation to an unconventional phenotype with tolerogenic features. J Immunol. (2011) 187:4129–39. 10.4049/jimmunol.1101243 21918189

[93] Oderup C LaJevic M Butcher EC. Canonical and noncanonical Wnt proteins program dendritic cell responses for tolerance. J Immunol. (2013) 190:6126–34. 10.4049/jimmunol.1203002 23677472 PMC3698971

[94] Manicassamy S Pulendran B. Dendritic cell control of tolerogenic responses. Immunol Rev. (2011) 241:206–27. 10.1111/j.1600-065X.2011.01015.x 21488899 PMC3094730

[95] Hong Y Manoharan I Suryawanshi A Majumdar T Angus-Hill ML Koni PA. β-catenin promotes regulatory T-cell responses in tumors by inducing vitamin A metabolism in dendritic cells. Cancer Res. (2015) 75:656–65. 10.1158/0008-5472.CAN-14-2377 25568183 PMC4333068

[96] Spranger S Bao R Gajewski TF. Melanoma-intrinsic β-catenin signalling prevents anti-tumour immunity. Nature. (2015) 523:231–5. 10.1038/nature14404 25970248

[97] Jiang J Lan C Li L Yang D Xia X Liao Q. A novel porcupine inhibitor blocks WNT pathways and attenuates cardiac hypertrophy. Biochim Biophys Acta Mol Basis Dis. (2018) 1864:3459–67. 10.1016/j.bbadis.2018.07.035 30076960

[98] Ho SY Keller TH. The use of porcupine inhibitors to target Wnt-driven cancers. Bioorg Med Chem Lett. (2015) 25:5472–6. 10.1016/j.bmcl.2015.10.032 26522946

[99] Waterman ML Lyou Y Habowski AN Chen GT. Inhibition of nuclear Wnt signalling: challenges of an elusive target for cancer therapy. Br J Pharmacol. (2017) 174:4589. 10.1111/bph.13963 28752891 PMC5727325

[100] Liu J Pan S Hsieh MH Ng N Sun F Wang T. Targeting Wnt-driven cancer through the inhibition of Porcupine by LGK974. Proc Natl Acad Sci USA. (2013) 110:20224–9. 10.1073/pnas.1314239110 24277854 PMC3864356

[101] You L He B Xu Z Uematsu K Mazieres J Fujii N. An anti-Wnt-2 monoclonal antibody induces apoptosis in malignant melanoma cells and inhibits tumor growth. Cancer Res. (2004) 64:5385–9. 10.1158/0008-5472.CAN-04-1227 15289346

[102] Mita MM Becerra C Richards DA Mita AC Shagisultanova E Osborne CRC Phase 1b study of WNT inhibitor vantictumab (VAN, human monoclonal antibody) with paclitaxel (P) in patients (pts) with 1st- to 3rd-line metastatic HER2-negative breast cancer (BC). J Clin Oncol. (2016) 34:2516 10.1200/JCO.2016.34.15_suppl.251627269942

[103] Davis SL Cardin DB Shahda S Lenz H-J Dotan E O'Neil B A phase Ib dose escalation study of vantictumab (VAN) in combination with nab-paclitaxel (Nab-P) and gemcitabine (G) in patients with previously untreated stage IV pancreatic cancer. J Clin Oncol. (2019) 37:249 10.1200/JCO.2019.37.4_suppl.249

[104] Jimeno A Gordon M Chugh R Messersmith W Mendelson D Dupont J. A first-in-human phase I study of the anticancer stem cell agent Ipafricept (OMP-54F28), a decoy receptor for Wnt ligands, in patients with advanced solid tumors. Clin Cancer Res. (2017) 23:7490–7. 10.1158/1078-0432.CCR-17-2157 28954784

[105] Fischer MM Cancilla B Yeung VP Cattaruzza F Chartier C Murriel CL. WNT antagonists exhibit unique combinatorial antitumor activity with taxanes by potentiating mitotic cell death. Sci Adv. (2017) 3:e1700090. 10.1126/sciadv.1700090 28691093 PMC5479655

[106] Kim MK. Novel insight into the function of tankyrase. Oncol Lett. (2018) 16:6895–902. 10.3892/ol.2018.9551 30546421 PMC6256358

[107] Haikarainen T Krauss S Lehtio L. Tankyrases: structure, function and therapeutic implications in cancer. Curr Pharm Des. (2014) 20:6472–88. 10.2174/1381612820666140630101525 24975604 PMC4262938

[108] Yang K Wang X Zhang H Wang Z Nan G Li Y. The evolving roles of canonical WNT signaling in stem cells and tumorigenesis: implications in targeted cancer therapies. Lab Investig. (2016) 96:116–36. 10.1038/labinvest.2015.144 26618721 PMC4731283

[109] Lee H-J Wang NX Shi D-L Zheng JJ. Sulindac inhibits canonical Wnt signaling by blocking the PDZ domain of the protein dishevelled. Angew Chemie Int Ed. (2009) 48:6448–52. 10.1002/anie.200902981 19637179 PMC2978498

[110] Fang L Zhu Q Neuenschwander M Specker E Wulf-Goldenberg A Weis WI. A small-molecule antagonist of the -Catenin/TCF4 interaction blocks the self-renewal of cancer stem cells and suppresses tumorigenesis. Cancer Res. (2016) 76:891–901. 10.1158/0008-5472.CAN-15-1519 26645562

[111] Ko AH Chiorean EG Kwak EL Lenz H-J Nadler PI Wood DL Final results of a phase Ib dose-escalation study of PRI-724, a CBP/beta-catenin modulator, plus gemcitabine (GEM) in patients with advanced pancreatic adenocarcinoma (APC) as second-line therapy after FOLFIRINOX or FOLFOX. J Clin Oncol. (2016) 34:e15721 10.1200/JCO.2016.34.15_suppl.e15721

[112] Canesin G Evans-Axelsson S Hellsten R Krzyzanowska A Prasad CP Bjartell A. Treatment with the WNT5A-mimicking peptide Foxy-5 effectively reduces the metastatic spread of WNT5A-low prostate cancer cells in an orthotopic mouse model. PLoS ONE. (2017) 12:e0184418. 10.1371/journal.pone.0184418 28886116 PMC5590932

[113] Säfholm A Leandersson K Dejmek J Nielsen CK Villoutreix BO Andersson T. A formylated hexapeptide ligand mimics the ability of Wnt-5a to impair migration of human breast epithelial cells. J Biol Chem. (2006) 281:2740–9. 10.1074/jbc.M508386200 16330545

[114] Piha-Paul SA Munster PN Hollebecque A Argilés G Dajani O Cheng JD. Results of a phase 1 trial combining ridaforolimus and MK-0752 in patients with advanced solid tumours. Eur J Cancer. (2015) 51:1865–73. 10.1016/j.ejca.2015.06.115 26199039 PMC5693226

[115] Schott AF Landis MD Dontu G Griffith KA Layman RM Krop I. Preclinical and clinical studies of gamma secretase inhibitors with docetaxel on human breast tumors. Clin Cancer Res. (2013) 19:1512–24. 10.1158/1078-0432.CCR-11-3326 23340294 PMC3602220

[116] Kummar S O'Sullivan Coyne G Do KT Turkbey B Meltzer PS Polley E. Clinical activity of the γ-secretase inhibitor PF-03084014 in adults with desmoid tumors (aggressive fibromatosis). J Clin Oncol. (2017) 35:1561–9. 10.1200/JCO.2016.71.1994 28350521 PMC5455706

[117] Atwood SX Whitson RJ Oro AE. Advanced treatment for basal cell carcinomas. Cold Spring Harb Perspect Med. (2014) 4:a013581. 10.1101/cshperspect.a013581 24985127 PMC4066644

[118] D'Amato C Rosa R Marciano R D'Amato V Formisano L Nappi L. Inhibition of Hedgehog signalling by NVP-LDE225 (Erismodegib) interferes with growth and invasion of human renal cell carcinoma cells. Br J Cancer. (2014) 111:1168–79. 10.1038/bjc.2014.421 25093491 PMC4453852

[119] Lee Y-R Chen M Lee JD Zhang J Lin S-Y Fu T-M. Reactivation of PTEN tumor suppressor for cancer treatment through inhibition of a MYC-WWP1 inhibitory pathway. Science. (2019) 364:eaau0159. 10.1126/science.aau0159 31097636 PMC7081834

[120] Persad A Venkateswaran G Hao L Garcia ME Yoon J Sidhu J. Active β-catenin is regulated by the PTEN/PI3 kinase pathway: a role for protein phosphatase PP2A. Genes Cancer. (2017) 7:368–82. 10.18632/genesandcancer.128. 28191283 PMC5302038

[121] Taye N Alam A Ghorai S Chatterji DG Parulekar A Mogare D. SMAR1 inhibits Wnt/β-catenin signaling and prevents colorectal cancer progression. Oncotarget. (2018) 9:21322–36. 10.18632/oncotarget.25093 29765542 PMC5940383

[122] Singh K Mogare D Giridharagopalan RO Gogiraju R Pande G Chattopadhyay S. p53 target gene SMAR1 is dysregulated in breast cancer: its role in cancer cell migration and invasion. PLoS ONE. (2007) 2:e660–75. 10.1371/journal.pone.0000660 17668048 PMC1924604

[123] Zhou A-D Diao L-T Xu H Xiao Z-D Li J-H Zhou H. β-Catenin/LEF1 transactivates the microRNA-371-373 cluster that modulates the Wnt/β-catenin-signaling pathway. Oncogene. (2012) 31:2968–78. 10.1038/onc.2011.461 22020335

[124] Mathai J Mittal SPKK Alam A Ranade P Mogare D Patel S. SMAR1 binds to T(C/G) repeat and inhibits tumor progression by regulating miR-371-373 cluster. Sci Rep. (2016) 6:33779. 10.1038/srep33779 27671416 PMC5037395

[125] Paul D Ghorai S Dinesh US Shetty P Chattopadhyay S Santra MK. Cdc20 directs proteasome-mediated degradation of the tumor suppressor SMAR1 in higher grades of cancer through the anaphase promoting complex. (2017) 8:e2882. 10.1038/cddis.2017.270 28617439 PMC5520925

